# The effect of age and body mass index on energy expenditure of critically ill medical patients

**DOI:** 10.1038/s41430-020-00747-8

**Published:** 2020-09-16

**Authors:** Christin Hölzel, Lorenz Weidhase, Sirak Petros

**Affiliations:** Medical ICU, University Hospital Leipzig, Leipzig, Germany

**Keywords:** Epidemiology, Nutrition

## Abstract

**Background:**

Data on the influence of age and body mass index (BMI) on energy metabolism of the critically ill are heterogeneous. Due to the increasingly aging critically ill population, investigation on age- and BMI-specific energy metabolism is relevant.

**Methods:**

A total of 394 indirect calorimetry measurements were conducted on 348 critically ill adult medical patients, including 46 repeat measurements after 3.6 ± 4.3 days. Measured resting energy expenditure (MREE) was compared for age groups, BMI, and gender. Predicted energy expenditure (PEE) using the Penn State, Swinamer, and Ireton-Jones equations and the ACCP recommendations was also compared with MREE.

**Results:**

The patients were 65.6 ± 14.5 years old. Their mean Acute Physiology and Chronic Health Evaluation II score was 27.6 ± 7.8. Mean BMI was 27.8 ± 8.4 kg/m^2^, and 25.6% were obese. MREE adjusted for ideal body weight decreased with increasing age, while it increased with increasing BMI. Age, BMI, and gender are independent determinants of MREE after adjusting for clinical factors (*R*^2^ = 0.34). All four prediction equations showed a proportional bias, with the Penn State equation performing acceptably. In 46 patients with repeat indirect calorimetry, there was no significant difference between the first and second MREE (*p* = 0.62).

**Conclusions:**

Age, BMI, and gender are independent determinants of resting energy expenditure in critically ill adults. Variations between measured and predicted energy expenditure are considerable. Should prediction equations be used, their performance in the specific population should be taken into consideration. Repeat indirect calorimetry may not always be necessary. However, this may depend on the length of stay and the extent of stress.

## Introduction

Defining the optimal energy requirement of the critically ill patient still remains a clinical challenge. Energy expenditure (EE) of the critically ill may vary depending on the extent of the metabolic stress as well as during the course of the disease [1, 2]. Therefore, measuring the EE of the critically ill may be favorable [3, 4]. The current European Society of Parenteral and Enteral Nutrition guideline recommends that EE should be measured using indirect calorimetry (IC) in mechanically ventilated patients and patients should receive 80–100% of the measured EE after day 3 [5]. The American Society of Parenteral and Enteral Nutrition guideline also favors the use of IC to determine EE. In the absence of IC, this guideline suggests to use either a predictive equation or a simplistic weight-based equation (25–30 kcal/kg/day) [6]. Since a calorimeter and trained personnel are not widely available, various prediction equations for calculation of EE have been widely used. However, the agreement between measured and predicted EE in critically ill patients is variable, depending on the formulae used and the type of critically ill study population [7–12].

The proportion of the aging population in the intensive care unit (ICU) is also continuously increasing [13–15]. This development may have a considerable influence on how predictive equations are to be applied in this ICU subpopulation. Physiologically, the metabolic rate decreases with increasing age. However, while the influence of age on EE has been studied in a large healthy population [16–18], publications on measured EE in the critically ill mostly included a relatively small subgroup of elderly patients. Furthermore, the epidemics of obesity may complicate the application of prediction equations [3].

The primary aim of the present study was to investigate the effect of age and body mass index (BMI) on EE of critically nonsurgical patients. The secondary aim was to compare measured resting energy expenditure (MREE) with that computed using commonly applied prediction equations, in order to determine if one of these equations is better suited in this patient population.

## Material and methods

This is a prospective observational study on EE of critically ill nonsurgical adult patients at the Medical ICU of the University Hospital of Leipzig. Data acquisition and analysis were carried out after approval by the local ethics board. IC is part of the routine clinical management of critically ill patients in the ICU. All variables reported in this study were collected as part of the standard patient care of the ICU. Informed consent was obtained from patients or their legal guardians regarding the use of the data on EE as well as other study variables for scientific purposes.

All critically ill patients on invasive ventilation were eligible for inclusion. Exclusion criteria were age <18 years, pregnancy and lactation, admission after a surgical procedure, brain death, invasive mechanical ventilation with a positive end-expiratory pressure (PEEP) > 12 cm H_2_O or fraction of inspiratory oxygen (FiO_2_) > 0.6, as well as refusal of the patient or the legal guardian regarding data processing for scientific purposes. Patients receiving macronutrient-containing solutions for reasons other than nutrition (for example 5% glucose solution for hypernatremia) were also excluded. Surgical patients were excluded because the ICU seldom admits such patients, and inclusion of such patients was considered a possible source of heterogeneity. IC was also carried out in patients on renal replacement therapy (RRT); however, these patients were excluded from this analysis because measurements without RRT were not systematically conducted on the same patients for appropriate comparison on possible influence of RRT on IC.

EE was measured using the Cosmed QUARK RMR (Cosmed, Rome, Italy). The calorimeter was calibrated every day before starting a measurement according to the instructions of the manufacturer. IC was carried out in the morning hours in fasting state, with feeding stopped at least 4 h prior to IC. Ventilator settings were left unchanged or no ventilator adjustments were done for at least 90 min prior to the commencement of the IC. The ICU uses as a rule norepinephrine as a vasopressor, sufentanil for opioid analgesia, and propofol for sedation. If a vasopressor, analgesia, or sedation was continuously administered, the dose was not changed immediately before and during IC. Should this be considered necessary by the care givers for clinical reasons, then IC was either not started or the results were discarded. Every IC measurement lasted at least 30 min, excluding the data for the first 5 min. Ambient temperature and humidity were recorded for every measurement. Patients were not disturbed during the IC, and no change made on ventilator variables. A measurement was excluded from further analysis if ventilator adjustments or any manipulation was necessary based on the decision of the ICU staff in charge of the patient. IC data were considered for further statistical analysis if the standard deviation for oxygen consumption (VO_2_) and carbon dioxide elimination (VCO_2_) was <10%.

The following data were collected for every patient: age, gender, actual body weight (ABW) and ideal body weight (IBW) (computed using the Hamwi equation [19]), BMI, major admission diagnosis on ICU admission, chronic underlying diseases (cardiac, pulmonary, hepatic, renal, neoplastic), Acute Physiology and Chronic Health Evaluation (APACHE) II score, Sequential Organ Function Assessment (SOFA) score on the day of IC, ventilation type (assisted or controlled), mean arterial blood pressure (MAP) at the start of the IC, body temperature (measured via urinary bladder catheter) during IC, maximum body temperature during the 24 h immediately before IC (Tmax), use of propofol, sufentanil, and norepinephrine during the IC. Regarding the three drugs, only their use (yes/no) was recorded rather than the dosage.

The following prediction equations for calculation of EE in critically ill patients were compared with MREE: Penn State 2003b for patients <60 years old with any BMI and those ≥60 years old and with BMI < 30 [8], Penn State 2010 for patients ≥60 years with BMI ≥ 30 [20], Swinamer [21], Ireton-Jones 1997 [22], and ACCP (American College of Chest Physicians) recommendations [23].

Statistical analysis was carried out with IBM SPSS version 24 (IBM, New York, USA). A descriptive analysis was performed, and numerical data tested for normal distribution using the Shapiro–Wilk and Kolmogorov–Smirnov tests. Numerical data are given as mean with standard deviation (with 95% confidence interval in brackets) for reasons of uniformity. The Student *t* test and one-way ANOVA were used for parametric variables, while the Mann–Whitney U test and Kruskal–Wallis test were applied for nonparametric variables. Categorical variables were analyzed using the chi-square test and Fisher’s exact test with two-tailed significance. Patients were a priori grouped into the following age groups and the data for MREE compared against each other using only the first IC measurement for every patient: ≤49, 50–59, 60–69, 70–79, and ≥80 years. Data were also analyzed for BMI groups (<25, 25.0–29.99, 30.0–39.99, and ≥40.0) as well as gender and compared against each other. Intergroup difference was analyzed using the Bonferroni test. A multiple linear regression analysis was conducted to determine the influence of the following variables on MREE adjusted for IBW (MREE_IBW_): age, gender, BMI, APACHE-II score, Tmax, the SOFA score, the type of invasive ventilation (assisted vs. controlled), and body temperature during the IC, as well as administration of norepinephrine, sufentanil, and propofol.

Bland–Altman plots are generated for the total study population to determine the agreement between MREE and predicted energy expenditure (PEE). In addition, absolute and proportional differences (predicted/measured × 100) between MREE and PEE are calculated for the age groups, BMI groups, and gender. The accuracy rates of every prediction equation, defined as a deviation from MREE of not more than ±10%, were also calculated for obese and nonobese patients stratified for age groups. Finally, in a subgroup of 46 patients with a second IC, the MREE between the first and second IC was compared. A *p* value < 0.05 was considered statistically significant.

## Results

We have conducted 684 IC measurements during the study period, out of which 115 (16.8%) were excluded because the standard deviations for VO_2_ and VCO_2_ were >10%. Out of the remaining 569 data sets, 111 measurements were excluded from the current analysis because IC was conducted while RRT was running. Another 64 IC data sets were excluded, because these were third or fourth measurements that would not have allowed a meaningful analysis due to their small number. The final analysis included 348 patients (225 males and 123 females) with 348 first-time and 46 repeat IC measurements, totaling 394 IC data sets.

The mean ambient temperature during IC was 23.1 ± 1.1 °C (22.9–23.3 °C) and the mean humidity was 41.0 ± 12.0% (38.7–43.3%). The mean standard deviation for VO_2_ and VCO_2_ was 5.2 ± 2.4% (5.0–5.4%).

The mean age of the patients was 65.6 ± 14.5 years (64.1–67.1), and 18.4% were 80 years or older. There was no statistically significant age difference between men and women (64.8 ± 14.6 vs. 66.9 ± 14.2 years, *p* = 0.44). The major admission diagnoses were infection (34.2%), cardiovascular diseases (24.4%), respiratory (18.7%), gastrointestinal (9.5%), metabolic (6.3%), neurological (2.6%), and other emergencies (4.3%). Demographic data classified for the predefined age groups are given in Table 1. The mean BMI of the total study population was 27.8 ± 8.4 (26.9–28.7) kg/m^2^, with 57.5% of them being overweight and 25.6% obese. The mean APACHE-II score for the total population was 27.6 ± 7.8 (26.8–28.4), and their mean SOFA score on the day of IC was 8.5 ± 3.9 (8.1–8.9). There was no significant difference between the age groups regarding gender distribution, the rate of obesity, the APACHE-II score excluding age points, the presence of at least one chronic underlying disease, as well as the SOFA score on the day of IC measurement.

**Table 1:** Demographic and clinical data of the study population classified for age groups.

	Age groups (years)				
	≤49	50–59	60–69	70–79	≥80
*N*	46	62	79	97	64
Age (years)*	39.3 ± 8.6 (36.7–41.8)	54.7 ± 2.8 (54.0–55.5)	64.1 ± 3.0 (63.4–64.8)	74.6 ± 2.9 (74.1–75.2)	83.1 ± 3.0 (82.4–83.8)
Males (%)	65.2%	71.0%	59.5%	71.1%	54.7%
BMI (kg/m^2^)^a^	26.7 ± 9.7 (23.8–29.6)	27.3 ± 7.7 (25.3–29.2)	30.6 ± 11.5 (28.0–33.2)	26.9 ± 5.1 (25.9–28.0)	26.6 ± 6.0 (25.1–28.1)
Obesity rate (%)	26.1%	25.8%	30.4%	24.7%	20.3%
APACHE-II score^b^	25.2 ± 7.5 (22.9–27.4)	25.9 ± 7.7 (23.9–27.9)	27.0 ± 7.8 (25.2–28.7)	29.0 ± 7.4 (27.5–30.5)	29.8 ± 7.8 (27.8–31.8)
APACHE-II excluding age points	24.4 ± 7.6 (22.1–26.7)	23.3 ± 7.7 (21.4–25.4)	23.1 ± 8.0 (21.3–24.9)	23.4 ± 7.5 (21.9–24.9)	23.8 ± 7.8 (21.8–25.8)
SOFA score	8.6 ± 4.2 (7.4–9.9)	8.7 ± 4.4 (7.6–9.8)	7.9 ± 3.6 (7.1–8.7)	8.4 ± 3.4 (7.7–9.1)	8.9 ± 4.0 (7.9–9.9)
≥1 chronic disease	50.0%	66.1%	64.6%	54.6%	50.0%

Figures in brackets are 95% confidence intervals.

*APACHE* Acute Physiology and Chronic Health Evaluation, *BMI* body mass index, *SOFA* Sequential Organ Function Assessment.

*p < 0.0001 for intergroup differences.

^a^*p* = 0.03 for age group 60–69 vs. 70–79 and *p* = 0.04 for age group 60–69 vs. ≥80.

^b^*p* = 0.02 for age group ≤49 vs. ≥80.

During the first IC, the MAP of the study patients was 78 ± 12.7 mmHg (76.6–79.3) and their body temperature was 37.1 ± 0.9 °C (37.0–37.2). Their maximum body temperature during the last 24 h before IC was 37.8 ± 1.0 °C (37.7–37.9). Prior to and during the IC, 45.1% of the patients were receiving norepinephrine and 69.3% were receiving propofol and sufentanil continuously. While 52.5% of the patients on propofol and sufentanil were also receiving norepinephrine, 82.7% of the patients on norepinephrine were also receiving propofol and sufentanil. Pressure support ventilation was the major of mode of ventilation (75%), while only 25% of the patients were on controlled mode of ventilation. Their mean respiratory quotient (RQ) was 0.78 ± 0.1 (0.77–0.79).

There was a decrease in MREE with increasing age, which was significant for the age group ≥80 years compared to the younger age groups. Taking MREE_IBW_, the difference remained significant between very old and younger male age groups, while we did not find a significant difference among female age groups (Table 2). There was no significant difference between males and females, except for total MREE in age groups 60–69 (*p* = 0.011) and 70–79 years (*p* = 0.001) and for MREE_IBW_ in the age group 60–69 years (*p* = 0.027). There was no significant difference for MREE between the major admission diagnoses (data not shown).

**Table 2:** Measured resting energy expenditure stratified for age groups and gender (*n* = 348).

	Age groups (years)				
Parameter	≤49	50–59	60–69	70–79	≥80
Males (*N*)	30	44	47	69	35
MREE (kcal/d)	2196 ± 845 (1881–2512)	2095 ± 607 (1910–2280)	2172 ± 531 (2016–2328)	1925 ± 518 (1801–2050)	1700 ± 408^*^ (1560–1841)
MREE_ABW_	27.9 ± 8.1 (24.9–30.9)	25.0 ± 9.2 (22.2–27.8)	24.8 ± 7.1 (22.7–26.9)	23.7 ± 6.7 (22.1–25.3)	22.5 ± 5.0 (20.8–24.2)^a^
MREE_IBW_	29.7 ± 9.7 (26.1–33.4)	27.5 ± 7.5 (25.2–29.7)	29.3 ± 7.9 (27.0–31.7)	26.1 ± 6.8 (24.5–27.8)	24.0 ± 5.9 (22.0–26.1)^b^
Females (*N*)	16	18	32	28	29
MREE (kcal/d)	1896 ± 643 (1554–2239)	1809 ± 598 (1511–2106)	1845 ± 576 (1636–2052)	1532 ± 398 (1378–1687)	1499 ± 492 (1312–1686)
MREE_ABW_	27.6 ± 12.5 (20.9–34.3)	25.6 ± 8.6 (21.4–29.9)	22.3 ± 6.6 (19.9–24.7)	22.3 ± 7.4 (19.4–25.1)	20.2 ± 5.3 (18.1–22.2)^a^
MREE_IBW_	33.9 ± 11.8 (27.6–40.0)	32.0 ± 9.9 (27.0–36.9)	34.4 ± 11 (30.5–38.3)	29.2 ± 7.9 (26.1–32.2)	27.4 ± 9.5 (23.8–31.0)

Figures in brackets are 95% confidence intervals.

*MREE* measured resting energy expenditure, *ABW* actual body weight, *IBW* ideal body weight.

*p < 0.05 compared to the first three age groups.

^a^*p* = 0.03 compared to the age group ≤49.

^b^*p* < 0.05 compared to age groups ≤49 and 60–69.

MREE stratified for BMI groups and gender is shown in Table 3. There was no significant age difference between the BMI groups. While MREE adjusted for actual body weight (MREE_ABW_) decreased with increasing BMI, MREE_IBW_ increased with increasing BMI. MREE_ABW_ was not significantly different between both genders throughout the BMI groups, while MREE_IBW_ was significantly higher in females than in males among those with BMI of 25–29.9 and those with BMI ≥ 40.

**Table 3:** Measured resting energy expenditure stratified for body mass index (BMI) groups and gender.

	BMI < 25.0		BMI 25.0–29.99		BMI 30.0–39.99		BMI ≥ 40	
Variable	Males (*n* = 103)	Females (*n* = 45)	Males (*n* = 72)	Females (*n* = 39)	Males (*n* = 37)	Females (*n* = 28)	Males (*n* = 13)	Females (*n* = 11)
Age (years)	63.9 ± 16.5	65.7 ± 14.9	66.9 ± 14.0	68.2 ± 13.8	65.1 ± 10.8	67.5 ± 15.2	59.9 ± 10.0	66.2 ± 10.9
Weight (kg)*	68.7 ± 9.6 (66.6–70.4)	56.7 ± 8.4 (54.1–59.2)	84.7 ± 7.8 (82.8–86.5)	73.8 ± 6.7 (71.6–76.0)	103.2 ± 12.6 (99.1–107.4)	89.8 ± 11.9 (85.1–94.4)	154.0 ± 32.6 (134.3–173.7)	133.4 ± 24.8 (116.7–150.0)
BMI*	22.1 ± 2.5	21.4 ± 2.5	27.0 ± 1.4	27.3 ± 1.5	33.6 ± 2.8	33.3 ± 2.7	50.5 ± 9.2	52.3 ± 8.2
MREE^a^ (kcal/d)	1871 ± 547 (1764–1978)	1473 ± 520 (1317–1629)	2030 ± 492 (1914–2146)	1724 ± 452 (1578–1871)	2140 ± 605 (1938–2342)	1753 ± 552 (1539–1967)	2646 ± 957 (2068–3224)	2333 ± 486 (2007–2660)
MREE_ABW_ (kcal/kg)^b^	27.3 ± 8.3 (25.7–28.9)	26.3 ± 10.1 (23.3–29.4)	23.9 ± 5.2 (22.6–25.2)	23.0 ± 5.8 (21.1–24.9)	20.9 ± 5.7 (19.0–22.8)	19.6 ± 5.7 (17.3–21.8)	17.2 ± 4.2 (14.6–19.7)	17.7 ± 3.2 (15.5–19.8)
MREE_IBW_ (kcal/kg)^a^	25.3 ± 7.1 (23.9–26.7)	27.4 ± 10.1 (24.4–30.4)	27.2 ± 6.3 (25.7–28.7)	30.9 ± 6.7 (28.7–33.1)	29.4 ± 7.9 (26.7–32.0)	31.8 ± 9.6 (28.0–35.5)	36.4 ± 10.5 (30.0–42.7)	45.7 ± 9.4 (39.4–52.0)

Figures in brackets are 95% confidence intervals.

*ABW* actual body weight, *IBW* ideal body weight, *MREE* measured resting energy expenditure in kcal/d.

*p < 0.0001 for differences between every group in both genders.

^a^*p* = 0.03 for BMI ≥ 40 vs. the other BMI groups in both genders.

^b^*p* = 0.007 for differences between every BMI group.

In the multivariate linear regression analysis to determine factors influencing MREE_IBW_, a significant regression equation was found (*p* < 0.0001) with a corrected *R*^2^ of 0.34. In the final analysis, age, female gender, and BMI are independent determinants of MREE_IBW_ after adjustment for various clinical factors (Table 4).

**Table 4:** Multivariate linear regression analysis of factors influencing measured energy expenditure adjusted for ideal body weight in critically ill medical patients.

Variable	Non-standardized regression coefficient B	95% CI		*p* value
		LL	UL	
Constant	−67.9	−101.4	−34.5	<0.0001
Age	−0.10	−0.15	−0.05	<0.0001
Female gender	4.05	2.53	5.57	<0.0001
BMI	0.47	0.39	0.56	<0.0001
Tmax	2.04	0.93	3.15	<0.0001
Analgesia/sedation	−2.05	−3.72	−0.37	0.017
APACHE-II score	0.07	−0.03	0.17	0.17
SOFA score	−0.05	−0.28	0.18	0.66
Temp_IC_	0.32	−0.87	1.51	0.59
Assisted ventilation	−0.16	−2.09	1.77	0.87
Vasopressor use	−0.97	−2.73	0.78	0.28

*APACHE* Acute Physiology and Chronic Health Evaluation, *BMI* body mass index, *Tmax* maximum body temperature during the last 24 h before indirect calorimetry, *Temp*_*IC*_ body temperature during indirect calorimetry, *LL* lower limit, *UL* upper limit.

**Fig. 1:**
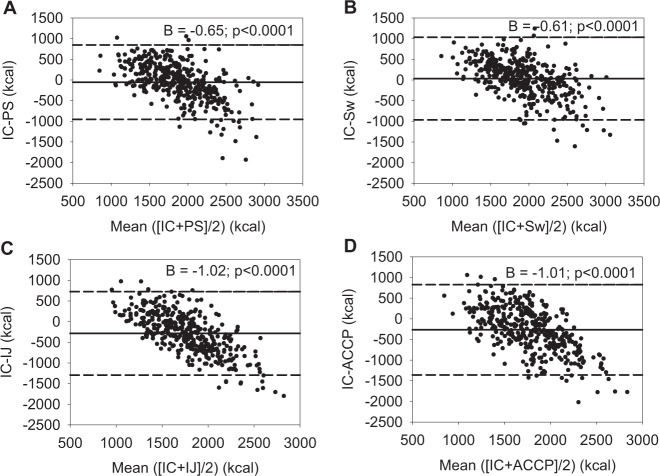
Bland-Altmann plots for agreements between MREE using indirect calorimetry (IC) and PEE using the four prediction equations taken into consideration. **a** MREE vs. PEE using the Penn State equation (PS); **b** MREE vs. PEE using the Swinamer equation (Sw); **c** MREE vs. PEE using the Ireton-Jones equation (IJ); **d** MREE vs. PEE using the ACCP recommendation (ACCP) (the value B within the figures is the unstandarized coefficient).

The Bland–Altman plots for agreements between measured EE and that computed using the prediction equations included are shown in Fig. 1a–d for the total study population. These demonstrate a proportional bias for all four prediction equations as shown by the unstandardized beta coefficient (*B*) given in the plots. Absolute and proportional deviations of PEE from MREE are given in Table 5 for age groups. The Penn State equation showed PEE within the predefined acceptable range of deviation for all age groups, while the Swinamer equation tended to generally overestimate EE. The Ireton-Jones equation underestimated EE with increasing age, while the ACCP recommendations underestimated EE in younger age groups. The Ireton-Jones equation and the ACCP recommendations generally underestimated EE in both genders (Supplementary File and Table 1). Both the Ireton-Jones equation and the ACCP recommendation also underestimated EE with increasing BMI (Supplementary File and Table 2). The total accuracy rates for the four prediction equations were: 34.8% for the Penn State equation, 33.5% for the Swinamer equation, 22.3% for the Ireton-Jones equation, and 21.6% for the ACCP recommendation (detailed accuracy rates stratified for age groups and obesity are given in Supplementary File and Table 3).

**Table 5:** Absolute (in kcal) and proportional deviation of predicted from measured energy expenditure classified for age groups (figures in brackets are 95% confidence intervals).

Age group	Penn State		Swinamer		Ireton-Jones		ACCP	
	Absolute	% MEE	Absolute	% MEE	Absolute	% MEE	Absolute	% MEE
≤49 years	−18 ± 580 (−181–145)	106.8 ± 28.0 (98.9–114.6)	52 ± 571 (−109–213)	110.3 ± 29.0 (102.2–118.4)	−146 ± 685 (−339–47)	102.0 ± 29.9 (93.4–110.4)	−456 ± 677 (−647 to −266)	84.3 ± 25.2 (77.2–91.3)
50–59 years	−100 ± 512 (−225–25)	101.5 ± 27.3 (95.0–108.1)	20 ± 501 (−103–142)	108.0 ± 28.6 (101.1–115.0)	−266 ± 560 (−403 to −130)	95.2 ± 32.2 (87.3–103.1)	−342 ± 566 (−480 to −205)	90.2 ± 29.7 (83.0–97.5)
60–69 years	−108 ± 441 (−201 to −15)	99.9 ± 24.1 (95.0–105.0)	21 ± 460 (−76–118)	106.5 ± 26.0 (101.0–112.0)	−374 ± 483 (−476 to −272)	87.4 ± 25.6 (82.0–92.8)	−396 ± 559 (−514 –278)	86.5 ± 26.3 (81.0–92.1)
70–79 years	−38 ± 436 (−119–44)	104.3 ± 30.2 (98.6–109.9)	52 ± 430 (−28–132)	109.3 ± 29.7 (103.8–114.9)	−292 ± 463 (−379 to −206)	90.3 ± 28.6 (85.0–95.6)	−158 ± 479 (−248 to −69)	97.8 ± 32.3 (91.8–103.8)
≥80 years	4 ± 365 (−81–88)	105.7 ± 23.4 (100.2–111.1)	29 ± 640 (−120–177)	108.7 ± 36.5 (100.3–117.2)	−277 ± 431 (−377 to −177)	89.4 ± 24.2 (83.8–95.0)	−74 ± 487 (−187–39)	103.3 ± 33.8 (95.5–111.2)

Finally, repeat IC measurements were conducted in 46 patients after a mean of 3.6 ± 4.3 (2.2–4.9) days. There was a significant difference between the first and the second measurement periods regarding the following variables: SOFA score 9.6 ± 4.0 vs. 7.4 ± 4.2 (*p* = 0.011), assisted mode of mechanical ventilation, 76.1% vs. 95.7% (*p* = 0.014); vasopressor requirement, 45.7% vs. 23.9% (*p* = 0.048); sedation, 76.1% vs. 52.2% (*p* = 0.029); on the other hand, there was no significant difference in MAP (77.5 ± 14.0 mmHg vs. 80.2 ± 14.8 mmHg, *p* = 0.38), body temperature during IC (37.3 ± 0.7 °C vs. 37.2 ± 0.8 °C, *p* = 0.49) and maximum body temperature during the last 24 h prior to IC (38.0 ± 0.9 °C vs. 38.1 ± 0.9 °C, *p* = 0.65). There was no significant difference between the first and second MREE (1929 ± 504 kcal/d vs. 1981 ± 476 kcal/d, *p* = 0.62) as well as the first and second RQ (0.78 ± 0.1 vs. 0.78 ± 0.1, *p* = 0.93).

## Discussion

This study on a large number of critically ill adult medical patients showed a decrease in measured EE with increasing age. Frankenfield has described in his recent detailed retrospective analysis of data from 826 critically ill patients that aging is associated with a nonlinear decrease in resting EE [24]. We have also observed that increasing BMI and female gender were independent determinants of an increase in EE adjusted for IBW after adjustment for several clinical factors. Our data regarding an increasing IBW-adjusted EE with increasing BMI are similar to the findings of others [25, 26]. Gender difference in EE has been reported in critically ill patients by Drolz et al. [27]. However, that study reported the opposite of what we have observed. The reason for this discrepancy is not clear. One should note that the independent factors included in our multiple linear regression as well in that from Drolz et al. explain only less than half of the variations observed (34% and 43.7%, respectively). One study on healthy volunteers did not find a significant difference in resting metabolic rate between men and women when adjusted for lean body mass, and fat mass explained a significant variation of resting metabolic rate in women [28]. A recent systematic review discussed several patient and clinical factors that influence EE [29]. This issue may explain the inconsistencies and contradictions regarding published results.

There was a proportional bias for the prediction equations included in our study. Several studies used accuracy rates to compare predicted with measured EE. We have also calculated these accuracy rates for the purpose of comparing our results with those from previous publications. We have found low accuracy rates for the prediction equations we have included in this study. The data from the literature are heterogeneous and at times contradictory. A retrospective analysis of 24-h IC data from predominantly surgical patients with a BMI < 30 kg/m^2^ and lesser disease severity than our patient population reported a better accuracy rate of 55% for Swinamer equation, 28% for Ireton-Jones equation, and 39% for Penn State equation [7]. Segadilha et al. reported on MREE in critically ill elderly patients and the significant deviation while applying predictive equations [30]. Kross et al. have also reported on the low accuracy of prediction equations in critically ill patients [11]. We have observed underestimation of EE among elderly patients with the Ireton-Jones equation, which was also reported by other authors [8]. In contrast, there was a general trend of overestimation using the Swinamer equation, similar to the findings of other groups [12, 31]. The Penn State equation showed the best approximation between PEE and MREE in our study population, which is similar to published validation studies [20, 32]. However, comparison between the studies is difficult, because the age stratification was not uniform and the critically ill population included was frequently heterogeneous. While we have found lower accuracy rates for Ireton-Jones equation among our obese patients, Glynn et al. [33] and Frankenfield et al. [8] reported a higher accuracy rate. The reason for these discrepancies could be the type of ICU population, and possibly the size of the study population too. Glynn et al. reported on only 25 critically ill patients with a BMI > 30 kg/m^2^, and data on disease severity were lacking, while Frankenfield et al. reported on 47 predominantly surgical patients. The Penn State equation fared best in our study population, which is similar to the findings of previous studies [12, 20, 34]. However, our accuracy rates are significantly lower than that reported by Frankenfield et al., who reported an accuracy rate of 76% using the Penn State equation in morbidly obese patients (BMI ≥ 45.0 kg/m^2^) [34]. One study in 44 obese patients reported an underestimation with the Harris–Benedict equation and an overestimation with the Ireton-Jones formula of the measured EE [35]. The Ireton-Jones equation and the ACCP recommendations underestimated REE in overweight and obese patients. Our results are consistent with prior findings that the ACCP recommendations are generally inadequate [11, 12, 30, 34]. Frankenfield has suggested new generation of potential prediction equations to better account for BMI extremes [24]. However, these equations have yet to be validated.

It is not yet clear how often IC should be performed in the critically ill and whether this would have any relevant clinical impact. Our limited data on repeat measurements in a subgroup of our study population imply that frequent IC may not be required. The issue on the frequency of IC measurements may also depend on the length of stay in the ICU and the clinical course during the ICU stay [36]. Therefore, our finding cannot be extrapolated to every critically ill patient.

Despite the critical issues regarding prediction equations, there are also limitations in applying IC, even if it would be broadly available [36]. First, the precision of the calorimeter and reproducibility of results could be variable. Second, ventilator requirements (high FiO_2_ and PEEP) or respiratory gas leak could be a hindrance. Third, critically ill patients are frequently restless, which may hamper a valid measurement. The true rate of missed or unsuccessful IC measurements is not systematically reported. In our study, IC measurements were discarded in 16.8% of the cases because the variations in the standard deviation for oxygen consumption and carbon dioxide elimination were greater than the predefined limit of 10%.

There are limitations to our study that should be considered in interpreting the data. It is a monocentric study, and the data represent the EE of severely ill medical patients. Therefore, one should be careful in extrapolating conclusions to other critically ill patients. However, these data on a large patient population with stratification for age groups, gender, and BMI may contribute to our understanding of measuring in contrast to predicting EE. Second, we have excluded patients on RRT, because we did not conduct IC with and without RRT in order to rule out the possible influence of RRT on gas exchange, particularly CO_2_. A recent pilot study on ten patients reported on a relevant CO_2_ elimination with continuous veno-venous hemofiltration [37]. However, its implication on IC has to be validated. Due to the considerably large proportion of critically ill patients on RRT [38], further EE studies with intra-individual comparisons are required.

In conclusion, age, gender, and BMI are independent predictors of resting EE in critically ill adult medical patients. EE adjusted for IBW decreased with increasing age, while it increased with increasing BMI. The Penn State equation performed best among the equations considered in this study, although with certain limitations. The Ireton-Jones equation and the ACCP recommendations for energy provision of the critically ill may be associated with a risk of underfeeding, particularly among overweight and obese patients.

## Supplementary information

Supplementary File
