# Correction to: The effect of age and body mass index on energy expenditure of critically ill medical patients

**DOI:** 10.1038/s41430-021-01030-0

**Published:** 2021-10-28

**Authors:** Christin Hölzel, Lorenz Weidhase, Sirak Petros

**Affiliations:** Medical ICU, University Hospital Leipzig, Leipzig, Germany

**Keywords:** Epidemiology, Nutrition

Correction to: *European Journal of Clinical Nutrition* 10.1038/s41430-020-00747-8 Published online 16 September 2020

The article The effect of age and body mass index on energy expenditure of critically ill medical patients, written by Christin Hölzel, Lorenz Weidhase, Sirak Petros was originally published Online First without Open Access. After publication in volume 75, issue 3, page 464–427 the author decided to opt for Open Choice and to make the article an Open Access publication. Therefore, the copyright of the article has been changed to © The Author(s) 2020 and the article is forthwith distributed under the terms of the Creative Commons Attribution 4.0 International License, which permits use, sharing, adaptation, distribution and reproduction in any medium or format, as long as you give appropriate credit to the original author(s) and the source, provide a link to the Creative Commons licence, and indicate if changes were made.

The images or other third party material in this article are included in the article’s Creative Commons licence, unless indicated otherwise in a credit line to the material. If material is not included in the article’s Creative Commons licence and your intended use is not permitted by statutory regulation or exceeds the permitted use, you will need to obtain permission directly from the copyright holder.

To view a copy of this licence, visit http://creativecommons.org/licenses/by/4.0/.

