# Supplementary material for: The effect of age and body mass index on energy expenditure of critically ill medical patients
Source: Eur J Clin Nutr. 2020 Sep 16;75(3):464–72. doi: 10.1038/s41430-020-00747-8 (PMC7493296; doi:10.1038/s41430-020-00747-8)
Supplement: Supplementary file 1 — Supplementary File [file 41430_2020_747_MOESM1_ESM.docx]

Supplementary Table 1. Gender-specific absolute (in kcal) and proportional deviations of predicted to measured energy expenditure

| gender | Penn State | | Swinamer | | Ireton Jones | | ACCP | |
| --- | --- | --- | --- | --- | --- | --- | --- | --- |
|  | absolute | % MEE | absolute | % MEE | absolute | % MEE | absolute | % MEE |
| males | -62±483  (-121- -2) | 102.9±26.8  (99.6-106.2) | 38±480  (-23-96) | 108.0±27.4  (104.7-111.4) | -282±534  (-348- -215) | 92.7±28.3  (89.2-96.2) | -232±566  (-302- -162) | 95.5±30.0  (91.8-99.2) |
| females | -39±414  (-109-30) | 104.3±27.1  (99.8-108.8) | 32±560  (-61-125) | 109.3±34.1  (103.4-114.9) | -290±475  (-369- -211) | 90.3±28.2  (85.6-95.0) | -327±539  (-416- -237) | 89.2±31.7  (83.9-94.5) |

Figures in brackets are 95% confidence intervals.

Supplementary Table 2. Absolute (in kcal) and proportional deviation of predicted from measured energy expenditure classified for body mass index (BMI) groups

| BMI | Penn State | | Swinamer | | Ireton Jones | | ACCP | |
| --- | --- | --- | --- | --- | --- | --- | --- | --- |
|  | absolute | % MEE | absolute | % MEE | absolute | % MEE | absolute | % MEE |
| <25.0 | -41±474  (113-30) | 105.3±31.4  (100.6-110.1) | 45±463  (-26-115) | 110.5±31.3  (105.8-115.3) | -195±502  (-271- -119) | 97.3±33.6  (92.2-102.4) | -141±529  (-222- -61) | 100.8±35.9  (95.3-106.2) |
| 25.0-29.99 | -52±373  (-118-15) | 101.5±20.4  (97.9-105.1) | 13±553  (-85-112) | 105.3±29.8  (100.0-110.6) | -340±452  (-420- -259) | 86.8±21.4  (83.0-90.6) | -239±446  (-319- -160) | 91.9±23.1  (87.8-96.0) |
| 30-39.99 | -49±525  (-169-71) | 104.3±27.0  (98.1-110.4) | 85±526  (-35-205) | 11.7±29.4  (105.0-118.4) | -298±552  (-425- -172) | 91.4±25.7  (85.5-97.2) | -349±558  (-477- -222) | 88.4±25.6  (82.6-94.2) |
| ≥40.0 | -157±540  (-375-61) | 97.1±20.2  (88.9-105.3) | -71±554  (-295-153) | 100.8±20.5  (92.5-109.1) | -565±642  (-824- -306) | 81.1±16.9  (74.2-87.9) | -959±694  (-1239- -679) | 64.9±16.8  (58.1-71.6) |

Figures in brackets are 95% confidence intervals

Supplement 3. Accuracy rates for prediction equations (percentage of estimates within ±10% of MEE) compared to measured energy expenditure

| Age group (years) | Penn State | Swinamer | Ireton Jones | ACCP |
| --- | --- | --- | --- | --- |
| ≤49 - obese  - non-obese | 28.6%  29.7% | 28.6%  35.1% | 21.4%  27.0% | 28.6%  21.6% |
| 50-59 - obese  - non-obese | 41.2%  42.0% | 29.4%  38.0% | 11.8%  28.0% | 17.6%  24.0% |
| 60-69 - obese  - non-obese | 39.3%  34.4% | 35.7%  37.7% | 21.4%  19.7% | 14.3%  19.7% |
| 70-79 - obese  - non-obese | 32.1%  32.9% | 28.6%  37.6% | 28.6%  25.9% | 17.9%  27.1% |
| ≥80 - obese  - non-obese | 20.0%  37.3% | 20.0%  25.4% | 6.7%  16.9% | 26.7%  16.9% |
